# Sustained Improvements in Antimicrobial Therapy and Clinical Outcomes following a Pharmacist-Led Antimicrobial Stewardship Intervention: Uncontrolled Before–After Study

**DOI:** 10.3390/jcm11030566

**Published:** 2022-01-23

**Authors:** Atsushi Uda, Kei Ebisawa, Hitomi Sakon, Mari Kusuki, Rie Izuta, Mariko Yahata, Ikuko Yano, Takayuki Miyara

**Affiliations:** 1 Department of Infection Control and Prevention, Kobe University Hospital, Kobe 650-0017, Japan; kansen@med.kobe-u.ac.jp (H.S.); hyakuta@med.kobe-u.ac.jp (M.K.); rizuta@med.kobe-u.ac.jp (R.I.); yahatapi@med.kobe-u.ac.jp (M.Y.); miyarat@med.kobe-u.ac.jp (T.M.); 2 Department of Pharmacy, Kobe University Hospital, Kobe 650-0017, Japan; iyano@med.kobe-u.ac.jp; 3 Department of Infectious Disease, Kobe University Hospital, Kobe 650-0017, Japan; k.f.ebisawa@gmail.com

**Keywords:** antimicrobial stewardship, blood culture collection, de-escalation therapy, antibiotic use, clinical outcome, infectious disease pharmacist

## Abstract

Our antimicrobial pharmacist-led intervention included: (a) a structured review of antibiotic prescriptions; (b) educating prescribers on antimicrobial therapy; (c) monthly reporting of department-level rates of blood sampling for culture. Daily review began in May 2018 and was discontinued after 10 months; however, the other interventions were conducted throughout the study period. This study aimed to evaluate the sustained impact of pharmacist’s interventions on antimicrobial therapy and clinical outcomes between the baseline (May–December 2017), intervention (May–December 2018), and post-intervention (May–December 2019) periods. The rate of blood culture collections before starting antipseudomonal agent therapy was significantly increased from the baseline to post-intervention periods (71% vs. 85%, *p* < 0.001). Antipseudomonal agent therapy was more frequently de-escalated in the post-intervention period than in the baseline period (73% vs. 54%, *p* = 0.038). Total use of antipseudomonal agents was reduced from the baseline to intervention periods and persisted during the post-intervention period (50.5 vs. 41.8 and 42.6 DDD per 1000 patient-days, *p* = 0.016 and *p* = 0.022, respectively). During the study period, there were significant reductions in the incidence of hospital-acquired *Clostridioides difficile* infection (1.12, 0.54, and 0.51 per 10,000 patient-days, respectively, *p* = 0.031) and 30-day mortality with bacteremia (19%, 18%, and 12%, respectively, *p* = 0.005). Our pharmacist-led interventions sustainably achieved appropriate antimicrobial therapy and improved clinical outcomes.

## 1. Introduction

Infectious diseases caused by pathogens having high antimicrobial resistance (AMR), leading to prolonged illness and high mortality, are a global health threat [1]. Antimicrobial stewardship programs (ASPs) foster appropriate antibiotic use, reduce the prevalence of AMR infections, and improve patient outcomes [2,3,4]. The Infectious Diseases Society of America guidelines recommend the implementation of ASPs in healthcare facilities [2]. Since 2010, we have conducted multidisciplinary prospective audits and feedback under the “Big Gun project” at our Kobe University Hospital [3,5]. Our previous report demonstrated that this project efficiently reduces the use of antipseudomonal agents and decreases the prevalence of methicillin-resistant *Staphylococcus aureus* (MRSA) [3]. However, since 2014, there have been few changes in the use of antipseudomonal agents and clinical outcomes under this project [3].

Infectious disease specialists recommend appropriate drugs to optimize therapy for patients in a broad range of infectious states. In April 2018, additional reimbursement for antimicrobial stewardship was introduced as a new medical fee in Japan; therefore, a new antimicrobial stewardship team (AST) was established to foster ASPs in our hospital. A full-time infectious disease pharmacist joined the team and started educational intervention and review of antimicrobial use to promote the appropriate use of broad-spectrum antibiotics. However, it remained unclear how the participation of a full-time pharmacist on AST affected antimicrobial therapy and patient outcomes. The aim of the present study was to evaluate the sustained effects of a pharmacist-led intervention on antibiotic use, the prevalence of resistant pathogens, and clinical outcomes.

## 2. Methods

### 2.1. Study Setting

This study was performed at the 934-bed tertiary care Kobe University Hospital in Japan. To avoid seasonal bias, all parameters were compared in the months of May–December using baseline (May–December 2017), intervention (May–December 2018), and post-intervention (May–December 2019) periods.

### 2.2. Antibiotics Available at the Hospital

The antibiotics available at our hospital are listed in Table 1. Antibiotics were categorized into antipseudomonal agents, anti-MRSA agents, and other antibiotics. In our hospital, broad-spectrum antibiotics were defined as antipseudomonal and anti-MRSA agents.

### 2.3. Infection Control Programs at the Hospital

Infection control programs at the hospital included medical staff education, hand hygiene promotion, environmental cleaning, contact precautions, and active surveillance of cultures. Contact precautions were taken with patients infected with multidrug-resistant organisms, such as MRSA, *Clostridioides difficile*, and *Pseudomonas aeruginosa*, which are resistant to more than one antibiotic among the three categories of antibiotics (carbapenems, fluoroquinolones, and aminoglycosides). Infection control programs remained constant throughout the study period.

### 2.4. Antimicrobial Stewardship Interventions by an Infectious Disease Pharmacist

#### 2.4.1. Pharmacist-Led Review of Patients Receiving Broad-Spectrum Antibiotics (Antipseudomonal Agents and Anti-MRSA Agents)

An infectious disease pharmacist in the AST began a daily review of antimicrobial use in May 2018 and continued it for the next 10 months. We reviewed the prescription of broad-spectrum antibiotics every weekday and contacted the prescribing physicians directly to optimize antibiotic use in cases of inappropriate prescriptions. The provided information consisted of appropriate microbiological culture collections, dose adjustment according to the patient’s renal function, choice of antibiotics based on microbiological data, adequate duration of antibiotic treatments, and performance of antimicrobial therapeutic drug monitoring. All broad-spectrum antibiotic prescriptions were reviewed during the intervention period.

#### 2.4.2. Educational Intervention for Promoting ASPs

Educational intervention began in June 2018 and was conducted until the end of the study period. The contents of lectures included problems associated with antibiotic consumption and AMR bacteria, the importance of collecting blood cultures, the role of the antimicrobial stewardship team, and rational antimicrobial strategies, such as appropriate choice of empirical and de-escalation therapy. Lectures were held for all hospital staff in June 2018 and February and June 2019, for representative physicians from each medical department in July 2018 and June 2019, and for the medical staff, including physicians, nurses, and pharmacists, in October and November 2018 and February 2019. In medical departments where blood cultures were seldom collected, lectures for the physicians were held in September 2018. Each lecture was held face-to-face to reach all prescribers.

#### 2.4.3. Monthly Reports of Department-Level Rates of Blood Culture Taking

Exchange of data on monthly blood culture rates from each department commenced in September 2018. This exchange was performed at monthly conferences attended by representative physicians from each department, and the data were disseminated to the prescribing physicians.

### 2.5. Outcomes

#### 2.5.1. Blood Culture Collections and De-Escalation Therapy

Blood culture collections before administering broad-spectrum antibiotics were defined as collections within 1 day before antibiotic treatment. We defined antibiotic de-escalation therapy as the discontinuation of at least one antibiotic or replacement of empirical broad-spectrum antibiotics with narrow-spectrum antibiotics based on positive blood culture results for bacterial infections. The rates of blood culture collections and de-escalation therapy were calculated based on the number of patients who received broad-spectrum antibiotics, except for those who were administered antibiotics prophylactically or who consulted infectious disease physicians.

#### 2.5.2. Antimicrobial Consumption (Dose and Duration)

The monthly antibiotic consumption in the hospital was expressed as the defined daily dose (DDD) per 1000 patient-days. The DDD was calculated using the Anatomical Therapeutic Chemical/DDD Index 2020 of the WHO Collaborating Center for Drug Statistics Methodology, and recorded as the median of each period. The duration of antibiotic treatment was the number of days between the start of the intravenous administration of an antibiotic class and the day in which the administration of the same class of antibiotic was discontinued. These parameters were analyzed based on the data pertaining to the antibiotics administered to the patients.

#### 2.5.3. Antimicrobial Expenses

The total cost for antibiotics was calculated by multiplying unit prices per dose by the number of total doses administered in each period. Drug prices as of April 2018 were used. All costs are presented in U.S. dollars ($); the U.S. dollar/yen exchange rate as of 1 December 2021 was 112.7 yen for 1 U.S. dollar.

#### 2.5.4. Clinical Outcomes

Hospital-acquired *C. difficile* infection (HA-CDI) was diagnosed in patients with *C. difficile* toxin production with diarrhea after 72 h of hospitalization. If diagnosed multiple times, patients were counted only the first time. The incidence of HA-CDI was summarized as cases per 10,000 patient-days. In cases of bacteremia, 30-day mortality was defined as death within 30 days after bacteremia onset. For patients with a history of two or more bacteremia episodes within 14 days, only the first episode was included in the analysis. Age, sex, and admission to the intensive care unit were evaluated on the date of the submission of blood cultures. The following bacterial species were defined as concomitants: *Bacillus* spp., *Corynebacterium* spp., *Propionibacterium* spp., *Micrococcus* spp., viridans group streptococci, and coagulase-negative staphylococci. If concomitant bacterial species were isolated from at least two different sets of blood drawn on the same day, these cases were defined as positive blood culture results and true bacteremia. The length of hospital stay by patients who received the antibiotics was the number of days between the date of admission and date of discharge.

#### 2.5.5. Microbiological Data

We obtained data on the susceptibility of *P. aeruginosa* from microbiology laboratory records, and the first isolate was used for analysis. The proportion of MRSA was calculated from the incidence of MRSA based on the number of patients with *S. aureus* in any culture. Data on these bacteria were collected as hospital-acquired infections after 72 h of hospitalization.

### 2.6. Statistical Analysis

Differences in the median of non-parametric variables were analyzed using the Kruskal–Wallis test followed by the Steel’s test for a post hoc comparison. Analyses of trends for categorical variables were performed using the Cochran–Armitage test. Results with a *p*-value < 0.05 were considered statistically significant. All parameters were analyzed using EZR on R commander (version 1.50) (Saitama Medical Center, Jichi Medical University, Saitama, Japan).

## 3. Results

### 3.1. Blood Culture Collections and De-Escalation Therapy

The rate of blood culture collections before initiating antibiotic use and the rate of de-escalation therapy are shown in Table 2. Before starting antipseudomonal agent therapy, the rate of blood culture collections was significantly increased from the baseline period (71%) to the post-intervention period (85%, *p* < 0.001). The rate of blood culture collections before anti-MRSA agents’ use did not differ throughout the study period (*p* = 0.072). Antipseudomonal and anti-MRSA agents were more frequently de-escalated to narrow-spectrum antibiotics in the post-intervention period than in the baseline period (*p* = 0.038 and *p* = 0.019, respectively).

### 3.2. Antibiotic Consumption

Table 3 shows the used amount of each class of antibiotic, and the monthly data are shown in Supplemental Table S1. The total use of antipseudomonal agents was significantly reduced from the baseline to the intervention period (50.5 vs. 41.8 DDD per 1000 patient-days, *p* = 0.016) and remained low during the post-intervention period (42.6 DDD per 1000 patient-days, *p* = 0.022). Carbapenem use was significantly reduced from the baseline to the intervention period by 23.4% (11.1 vs. 8.5 DDD per 1000 patient-days, *p* = 0.004) and from the baseline to the post-intervention period by 27% (8.1 DDD per 1000 patient-days, *p* = 0.022). The use of antipseudomonal fourth-generation cephalosporins and aminoglycosides was significantly reduced from the baseline to the post-intervention period (*p* = 0.022 and *p* = 0.029, respectively). The use of fluoroquinolones significantly decreased from the baseline to the intervention period (4.4 vs. 3.1 DDD per 1000 patient-days, *p* = 0.006) but did not differ between the baseline and post-intervention periods (*p* = 0.24). The use of first-generation cephalosporins was similar between the baseline and intervention periods (*p* = 0.95) but significantly reduced from the baseline to the post-intervention period (38.9 vs. 4.1 DDD per 1000 patient-days, *p* = 0.001). There were significant increases from the baseline to the post-intervention period in the use of penicillins except for antipseudomonal agents (36.3 vs. 47 DDD per 1000 patient-days, *p* = 0.006), second-generation cephalosporins (12.6 vs. 28.4 DDD per 1000 patient-days, *p* = 0.002), and third-generation cephalosporins except for antipseudomonal agents (9.5 vs. 11.5 DDD per 1000 patient-days, *p* = 0.025). The use of other antibiotics showed no significant differences during the study period. 

Table 4 shows the median duration of each antibiotic treatment. The monthly median and mean durations of antibiotic treatment are presented in Supplemental Table S2 and Table S3, respectively. The duration of antipseudomonal agent treatments was significantly shortened from the baseline to the intervention period (6 vs. 5 days, *p* = 0.001) and remained short during the post-intervention period (5 days, *p* = 0.007). There were no significant changes in the duration of treatments with anti-MRSA agents during the study period.

Table 5 shows the total cost of antibiotic injection in each period, and monthly data are shown in Supplemental Table S4. Compared with those at the baseline, antibiotic costs were reduced by USD 57,210 in the intervention period and USD 49,179 in the post-intervention period.

### 3.3. Susceptibility of P. aeruginosa and the Proportion of MRSA among all S. aureus Isolates

Table 6 shows the susceptibility of *P. aeruginosa* isolates to specific antibiotics. Supplemental Table S5 shows the susceptibility results of *P. aeruginosa* to each antibiotic, presented as susceptible/intermediate/resistant (S/I/R), on a monthly basis. There were no significant changes in the susceptibility to any antibiotics throughout the study period.

The proportion of MRSA among all isolates is presented in Table 7. There were no significant differences in the occurrence of MRSA among the three periods (*p* = 0.32). 

### 3.4. Clinical Outcomes

**Figure 1:**
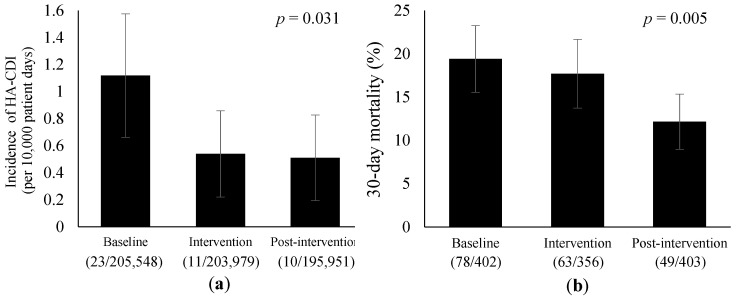
Trends in the clinical outcomes before and after the interventions. Error bars represent 95% confidence intervals. (**a**) Incidence of HA-CDI per 10,000 patient-days. HA-CDI, hospital-acquired *Clostridioides difficile* infection. (**b**) 30-day mortality due to bacteremia.

The clinical outcomes before and after the interventions are shown in Figure 1 and Table 8. The incidence of HA-CDI was significantly reduced from the baseline to the post-intervention period (1.12 vs. 0.51 cases per 10,000 patient-days, *p* = 0.031). The 30-day mortality showed a significant reduction from 19% at the baseline period to 12% during the post-intervention period (*p* = 0.005). Supplemental Table S6 shows the demographic characteristics of the patients with bacteremia. No significant differences were observed among the three periods.

Table 8 shows the length of hospital stay of patients undergoing antibiotic treatment. The monthly median and mean data are presented in Supplemental Table S7 and Table S8, respectively. Among patients who received different classes of antibiotics, no significant difference in the length of hospitalization stay during the study period was observed.

## 4. Discussion

The Japanese Ministry of Health, Labour and Welfare revised medical fees for the implementation of AST in 2018 to foster ASPs. In our hospital, a full-time infectious disease pharmacist joined the team and promoted appropriate antimicrobial therapy through the review of antimicrobial use and educational intervention. The review was conducted daily during the intervention period and discontinued during the post-intervention period. The purpose of this study was to evaluate the sustained effects of pharmacist-led interventions on antimicrobial therapy, microbiological data, and patient outcomes. We found significant increasing trends in the rate of blood culture collections and the rate of de-escalation therapy throughout the study period. Furthermore, the antibiotic use and the incidence of HA-CDI were reduced, with an improved survival rate after the interventions. These effects were sustained even during the post-intervention period. To the best of our knowledge, this is the first study of its kind to demonstrate that pharmacist-led interventions on AST improved the antimicrobial therapy and clinical outcomes since the revision of reimbursement for AST implementation in Japan.

To optimize early antimicrobial therapy, we reviewed patients who were administered broad-spectrum antibiotics and, when inappropriate prescriptions were found, flagged them with physicians to promote optimal use. In the present study, there were significant increases in the rate of blood culture collections before administering antipseudomonal agents and the rate of de-escalation therapy. The 2012 UK Surviving Sepsis Campaign recommends de-escalation therapy, empirically changing from broad-spectrum antibiotics to narrow-spectrum antibiotics based on microbiological findings in blood cultures prior to initiating antimicrobial therapy [6]. Increased blood culture collections and de-escalation therapy are protective strategies associated with lower mortality [7,8,9,10]. Consistent with these reports, there was a significant reduction in 30-day mortality due to bacteremia during our study period. This indicates that our intervention leads to better patient outcomes. Moreover, daily review of the medical record of each patient is a great burden for specialists; however, we observed a sustained reduction in the use of antipseudomonal agents and improved clinical outcomes even after daily review was discontinued. An educational antimicrobial stewardship program has a long-term effect on the reduction of antibiotic consumption and increase of appropriate antimicrobial therapy [11]. The educational interventions that were implemented throughout the study period may have contributed to our sustained benefits. As healthcare facilities have finite resources and a limited number of clinical pharmacists specializing in ASPs in Japan, our interventions could be effective strategies, especially in hospitals with limited resources.

A previous systematic review has reported that the rate of reduction in restricted antibiotic consumption after the implementation of ASPs is 26.6%, and that in carbapenem consumption this is 18.5% based on 26 studies from around the world [12]. Although the use of carbapenems before interventions in our hospital was markedly lower than that in most other national university hospitals in Japan [13], the total use of antipseudomonal agents was reduced by 15.6–17.2% and that of carbapenems by 23.4–27%. This may be because empirical broad-spectrum antibiotics were more frequently changed to narrow-spectrum antibiotics based on increased blood culture collections. These findings are also supported by other results showing that the duration of antipseudomonal agent treatment is shortened after interventions. According to a previous study [14], ASPs reduce antimicrobial treatment costs after implementation. Our study showed savings in the total antibiotic cost during the intervention and post-intervention periods, with a reduction in antibiotic consumption.

The extensive use of broad-spectrum antibiotics may be a risk factor for the development of resistant pathogens [15]. Many studies have reported that antipseudomonal agent use and previous exposure are correlated with changes in *P. aeruginosa* susceptibility patterns [16,17,18]. In Japan, the susceptibility to amikacin is over 95%, and that of other classes is 80% [19]. In our study, the susceptibility of *P. aeruginosa* to each antibiotic remained at approximately 90% throughout the study period. Our rational antimicrobial strategy to reduce the consumption of antipseudomonal agents may have contributed to preventing the development of resistance in *P. aeruginosa*. Nevertheless, although the rate of de-escalation therapy with anti-MRSA agents increased during the study period, there were no significant differences in the use of anti-MRSA agents or the proportion of MRSA among all *S. aureus* isolates. This may be related to the small sample size of the anti-MRSA agent group.

*C. difficile* is the leading cause of life-threatening infectious diarrhea in hospitals. Broad-spectrum antibiotics are likely to disrupt the normal gut microbiota and increase the risk of contracting CDI. Among antipseudomonal agents, carbapenems and fluoroquinolones have been identified as particularly high-risk factors for acquiring CDI [20,21]. Restrictions on the use of antipseudomonal agents, associated with a high risk of CDI, lead to a reduction in CDI in hospitalized patients [22,23]. A recent systematic literature review reported that the prevalence of CDI in Japan is 0.8–4.7 cases/10,000 patient-days [24], which is lower than that reported in Europe and the United States [25,26,27]. The incidence of HA-CDI in our hospital before intervention was lower than that in these previous reports, and we found a further reduction after the implementation of our intervention. There was a significant reduction in the use of these high-risk antibiotics, which may have contributed to the lower incidence of HA-CDI. The prevalence of HA-CDI is also controlled by infection control programs consisting of medical staff education, hand hygiene promotion, environmental cleaning, contact precautions, and active surveillance of cultures [28]. Conducting infection control programs together with ASPs could prevent the spread of resistant pathogens effectively.

Owing to a national shortage of cefazolin in Japan, the prescription of cefazoline has been restricted in our hospital since March 2019 [29]. Therefore, the use of first-generation cephalosporins significantly decreased during the post-intervention period. In contrast, there was a significant increase in the use of non-antipseudomonal penicillins, second-generation cephalosporins, and non-antipseudomonal third-generation cephalosporins between the baseline and post-intervention periods. These agents and broad-spectrum antibiotics, such as fluoroquinolones and anti-MRSA agents, are listed as alternative antimicrobials for cefazolin by the Ministry of Health, Labour and Welfare of Japan [30]; however, in the present study, we had a rational antimicrobial strategy for treating bacteremia due to methicillin-susceptible *S. aureus* [29], which may prevent an increase in the use of broad-spectrum antibiotics.

This study has some limitations. First, we conducted a retrospective observational study with a small sample size at a single university hospital in Japan. Therefore, multicenter studies with a larger sample size are required. Second, because we reviewed the data extracted from medical electronic records, measurement bias was inevitable. Third, the study period could be too short to evaluate whether the intervention had an impact on AMR. Although our benefits were sustained in over 10 months after the intervention was discontinued, Gerber et al. have shown that the effects of ASPs are lost within 18 months after the withdrawal of the intervention [31]. However, since the beginning of 2020, the unprecedented coronavirus disease 2019 pandemic has had a drastic effect on antibiotic usage [32,33], leading to difficulty in accurately assessing our intervention over a longer period of time. Fourth, while monitoring the rates of blood culture collections and de-escalation therapy, we excluded patients who were administered antibiotics prophylactically and those who consulted infectious disease physicians. Therefore, we could not examine all patients in these parameters. Lastly, we continued to implement other interventions, such as weekly audit and feedback programs through this study period [3,5]. We may not have sufficiently evaluated the effects of these on our study.

## 5. Conclusions

An infectious disease pharmacist-led intervention contributed to a continuous increase in the rate of blood culture collections before starting broad-spectrum antibiotics and the rate of de-escalation therapy, which may have led to a reduction in the total use of antipseudomonal agents and incidence of HA-CDI. Furthermore, the survival rate of patients with bacteremia improved during the study period. The analyses of our results suggest that continuous educational intervention and review of antimicrobial use are effective in optimizing antimicrobial therapy and improving clinical outcomes, which were sustained after the discontinuation of review intervention. The number of infectious disease specialists on antimicrobial stewardship is limited in healthcare settings; hence, these efficient and sustainable interventions should be more widely implemented as supporting strategies for ASPs.

## Figures and Tables

**Table 1:** Classification of antibiotics available at Kobe University Hospital.

Classes	Antibiotics
Antipseudomonal agents	
Antipseudomonal penicillins	Piperacillin and piperacillin/tazobactam
Antipseudomonal third-generation cephalosporins	Ceftazidime
Antipseudomonal fourth-generation cephalosporins	Cefepime and cefozopran
Monobactams	Aztreonam
Carbapenems	Meropenem and doripenem
Fluoroquinolones	Ciprofloxacin, levofloxacin, and pazufloxacin
Aminoglycosides	Amikacin, tobramycin, and gentamicin
Polymyxins	Colistin
Anti-MRSA agents	Vancomycin, teicoplanin, daptomycin, and linezolid
Other antibiotics	
Penicillins except for antipseudomonal agents	Benzylpenicillin, ampicillin, and ampicillin/sulbactam
First-generation cephalosporins	Cefazolin
Second-generation cephalosporins	Cefotiam, cefmetazole, and flomoxef
Third-generation cephalosporins except for antipseudomonal agents	Ceftriaxone and cefotaxime
Other non-antipseudomonal agents	Azithromycin, clindamycin, fosfomycin, minocycline, and metronidazole

**Table 2:** Rate of blood culture collections before initial antibiotic use and de-escalation therapy.

	Baseline	Intervention	Post-Intervention	*p*
Blood culture collections before antibiotic use, *n* (%)				
Antipseudomonal agents	539/758 (71)	563/681 (83)	596/698 (85)	<0.001
Anti-MRSA agents	76/95 (80)	66/83 (80)	69/76 (91)	0.072
Antimicrobial de-escalation therapy, *n* (%)				
Antipseudomonal agents	33/61 (54)	62/81 (77)	52/71 (73)	0.038
Anti-MRSA agents	7/12 (58)	16/16 (100)	10/10 (100)	0.019

MRSA, methicillin-resistant *Staphylococcus aureus*.

**Table 3:** Defined Daily Dose (DDD) of antibiotics per 1000 patient-days, median (IQR).

	Baseline	Intervention	Post-Intervention	*p*-Value Baseline vs. Intervention	*p*-Value Baseline vs. Post-Intervention
Antipseudomonal agents	50.5 (47.4–55.4)	41.8 (37.0–45.8)	42.6 (42.2–44.1)	0.016	0.022
Antipseudomonal penicillins	19.5 (16.5–20.5)	18.2 (15.5–19.9)	19.9 (18.8–20.9)	0.68	0.72
Antipseudomonal third-generation cephalosporins	1.8 (0.9–2.1)	1.3 (1–1.5)	2 (1.5–2.3)	0.47	0.61
Antipseudomonal fourth-generation cephalosporins	10.9 (9.5–13.4)	8.8 (8–9.4)	8.1 (6.2–9.1)	0.083	0.022
Monobactams	0.0 (0–0.1)	0.0 (0–0.1)	0.0 (0–0)	0.71	1
Carbapenems	11.1 (10.4–11.3)	8.5 (7.7–9.9)	8.1 (6.8–9.5)	0.004	0.022
Fluoroquinolones	4.4 (4.1–4.8)	3.1 (2.6–3.5)	3.5 (3.3–4.7)	0.006	0.24
Aminoglycosides	2.1 (1.3–2.6)	1.1 (0.9–1.4)	0.8 (0.7–1.2)	0.073	0.029
Polymyxins	0.1 (0–0.7)	0.2 (0.1–0.3)	0 (0–0)	0.98	0.17
Anti-MRSA agents	19.4 (17.3–21.5)	20.3 (17.7–22.4)	21.3 (19.2–23)	0.97	0.16
Other antibiotics	101.3 (99.6–109)	108 (103.3–112)	94.8 (91–101.7)	0.44	0.2
Penicillins except for antipseudomonal agents	36.3 (35.8–39.5)	40.8 (35.2–44.8)	47 (43.8–50.7)	0.65	0.006
First-generation cephalosporins	38.9 (37.3–40.4)	39.5 (36.2–41.9)	4.1 (3.8–5.2)	0.95	0.001
Second-generation cephalosporins	12.6 (11.6–13.8)	11.7 (10.5–12.6)	28.4 (27.4–28.9)	0.61	0.002
Third-generation cephalosporins except for antipseudomonal agents	9.5 (8.2–9.8)	9.5 (9.1–11.3)	11.5 (10.7–12.9)	0.88	0.025
Other non-antipseudomonal agents	3.9 (3.4–4.7)	6.2 (4.5–8.1)	3.9 (3.4–5.4)	0.15	1
Total	175.4 (167.4–178.3)	171.3 (160.4–179.4)	157.6 (154–163.9)	0.97	0.051

IQR, interquartile range; MRSA, methicillin-resistant *Staphylococcus aureus*.

**Table 4:** Duration of each antibiotic treatment, median days (IQR).

	Baseline		Intervention		Post-Intervention		*p*-Value Baseline vs. Intervention	*p*-Value Baseline vs. Post-Intervention
	*n*	Median (IQR)	*n*	Median (IQR)	*n*	Median (IQR)		
Antipseudomonal agents	1689	6 (3–8)	1562	5 (3–8)	1559	5 (3–8)	0.001	0.007
Anti-MRSA agents	680	4 (1–9)	701	4 (1–8)	727	3 (1–8)	0.080	0.12
Other antibiotics	7414	2 (1–4)	7367	3 (1–4)	7302	2 (1–4)	0.097	<0.001

IQR, interquartile range; MRSA, methicillin-resistant *Staphylococcus aureus*.

**Table 5:** Antibiotic cost (USD).

	Baseline	Intervention	Post-Intervention
Total cost of antibiotics	649,165	591,955	599,986
Antibiotic cost saving		57,210	49,179

**Table 6:** Susceptibility of *Pseudomonas aeruginosa* to each antibiotic, *n* (%).

	Baseline (*n* = 112)	Intervention (*n* = 106)	Post-Intervention (*n* = 135)	*p*
Piperacillin	100 (89)	95 (90)	122 (90)	0.78
Cefepime	104 (93)	97 (92)	122 (90)	0.49
Meropenem	99 (88)	99 (93)	126 (93)	0.17
Amikacin	112 (100)	106 (100)	132 (98)	0.051
Levofloxacin	103 (92)	101 (95)	128 (95)	0.23

**Table 7:** Proportion of MRSA among all isolates of *Staphylococcus aureus*.

	Baseline	Intervention	Post-Intervention	*p*
MRSA, *n* (%)	111/179 (62)	132/217 (61)	167/290 (58)	0.32

MRSA, methicillin-resistant *Staphylococcus aureus*.

**Table 8:** Length of hospital stay by patients who received antibiotics, median days (IQR).

	Baseline	Intervention	Post-Intervention	*p*-Value Baseline vs. Intervention	*p*-Value Baseline vs. Post-Intervention
Antipseudomonal agents	29 (15–54)	31 (16–56)	27 (14–49)	0.41	0.17
Anti-MRSA agents	42 (24–75)	43 (25–73)	44 (25–73)	0.85	0.93
Other antibiotics	12 (6–23)	12 (6–23)	12 (6–22)	0.92	0.11

MRSA, methicillin-resistant *Staphylococcus aureus*.

## Data Availability

Not applicable.

## Supplementary Materials

The following are available online at https://www.mdpi.com/article/10.3390/jcm11030566/s1, Table S1: Monthly Defined Daily Dose (DDD) of antibiotics per 1000 patient-days; Table S2: Monthly duration of each antibiotic treatment, median days (IQR); Table S3: Monthly duration of antimicrobial therapy, mean days (SD); Table S4: Monthly antibiotic cost (US$); Table S5: Susceptibility categorization (S/I/R) of *P. aeruginosa* to each antibiotic per month; Table S6: Demographic characteristics of patients with bacteremia; Table S7: Monthly length of hospital stay by patients who received antibiotics, median days (IQR); Table S8: Monthly length of hospital stay by patients who received antibiotics, mean days (SD).
