# Supplementary material for: Sustained Improvements in Antimicrobial Therapy and Clinical Outcomes following a Pharmacist-Led Antimicrobial Stewardship Intervention: Uncontrolled Before–After Study
Source: J Clin Med. 2022 Jan 23;11(3):566. doi: 10.3390/jcm11030566 (PMC8837014; doi:10.3390/jcm11030566)
Supplement: Supplementary file 1 [file jcm-11-00566-s001.zip › jcm-1485185-supplementary.pdf]

Supplemental Table S1. Monthly Defined Daily Dose (DDD) of antibiotics per 1,000 patient-days

	May-17	Jun-17	Jul-17	Aug-17	Sep-17	Oct-17	Nov-17	Dec-17	May-18	Jun-18	Jul-18	Aug-18
Antipseudomonal agents	57.2	55.4	55.4	46.1	48.5	52.4	47.9	39.3	37.6	35.2	33.1	45.5
Antipseudomonal penicillins	16.5	21.3	19.6	16.5	20.2	22.7	19.4	15.3	18.8	15.7	14.8	19.4
Antipseudomonal third generation cephalosporins	4.5	2.1	2.1	1.0	2.0	0.7	0.1	1.6	0.2	1.0	1.2	1.7
Antipseudomonal fourth generation cephalosporins	15.8	13.6	13.3	11.7	10.0	9.4	9.6	7.1	6.7	8.3	8.0	10.9
Monobactams	0.0	0.0	0.0	0.1	0.0	0.0	0.0	0.0	0.0	0.0	0.1	0.0
Carbapenems	10.3	11.6	11.2	10.5	11.1	13.5	11.1	9.6	7.8	7.8	5.5	7.5
Fluoroquinolones	5.7	3.8	4.5	4.3	4.2	3.7	6.2	4.5	3.0	1.2	2.5	3.9
Aminoglycosides	3.3	2.2	4.1	2.0	0.9	2.4	1.4	1.0	0.9	1.0	0.9	1.8
Polymyxins	1.0	0.7	0.7	0.0	0.0	0.0	0.1	0.0	0.2	0.1	0.0	0.4
Anti-MRSA agents	20.8	22.6	22.3	21.2	17.6	18.0	16.6	15.3	19.1	11.4	13.4	20.1
Other antibiotics	100	97.6	102.1	100.5	109.0	108.8	98.2	111.7	106.8	93.3	104.8	98.9
Penicillins except for antipseudomonal agents	36.1	34.0	39.5	35.3	36.4	46.3	35.9	39.7	41.7	34.0	33.2	35.6
First generation cephalosporins	39.1	37.0	37.3	38.9	44.2	37.3	38.9	46.6	33.2	34.6	43.1	41.7
Second generation cephalosporins	9.6	12.6	14.2	12.3	15.1	13.7	9.4	12.6	9.5	11.6	10.7	9.9
Third generation cephalosporins except for antipseudomonal agents	9.8	7.8	8.3	10.4	9.5	7.6	9.5	9.8	16.2	9.7	9.5	7.0
Other non-antipseudomonal agents	5.6	6.2	2.8	3.5	3.8	3.9	4.5	3.0	6.2	3.4	8.4	4.8
Total	178	175.6	179.8	167.8	175.1	179.2	162.7	166.3	163.5	139.8	151.3	164.5

MRSA, methicillin-resistant *Staphylococcus aureus*

Supplemental Table S1. *Continued*

	Sep-18	Oct-18	Nov-18	Dec-18	May-19	Jun-19	Jul-19	Aug-19	Sep-19	Oct-19	Nov-19	Dec-19
Antipseudomonal agents	47.8	43.0	40.6	46.8	42.4	42.1	46.7	43.8	42.2	45.0	37.0	42.9
Antipseudomonal penicillins	24.6	17.6	14.8	21.3	22.7	21.2	18.0	19.0	20.8	20.0	16.9	19.8
Antipseudomonal third generation cephalosporins	1.4	0.7	1.8	1.5	1.5	1.7	2.3	2.2	2.6	2.5	0.9	1.5
Antipseudomonal fourth generation cephalosporins	8.0	10.1	9.2	9.2	5.1	6.5	9.2	10.6	8.0	9.0	5.2	8.2
Monobactams	0.0	0.0	0.0	0.3	0.0	0.0	0.1	0.0	0.0	0.0	0.0	0.0
Carbapenems	10.0	9.2	10.2	9.8	9.3	6.9	12.1	6.7	6.8	6.4	9.9	9.4
Fluoroquinolones	3.3	3.9	3.2	2.6	3.0	4.7	3.6	4.7	3.3	5.4	3.5	2.7
Aminoglycosides	0.5	1.3	1.2	1.5	0.7	1.2	1.4	0.5	0.7	1.7	0.6	1.0
Polymyxins	0.0	0.2	0.2	0.5	0.0	0.0	0.0	0.0	0.0	0.0	0.0	0.3
Anti-MRSA agents	23.2	22.1	24.8	20.6	21.3	24.0	22.9	21.3	18.7	18.5	19.4	23.4
Other antibiotics	109.2	135.6	112.8	111.7	89.8	91.4	112.6	102.9	93.4	89.3	101.3	96.3
Penicillins except for antipseudomonal agents	43.6	51.6	48.6	39.9	44.7	43.1	54.9	52.9	44.1	41.5	49.4	50.0
First generation cephalosporins	36.8	39.1	39.8	42.7	4.2	4.0	3.4	4.3	3.5	9.0	8.0	4.1
Second generation cephalosporins	13.2	15.0	12.4	11.8	25.7	28.1	31.2	29.5	28.7	28.8	27.8	26.5
Third generation cephalosporins except for antipseudomonal agents	9.6	21.6	8.9	9.2	11.7	13.5	17.5	10.9	10.4	6.8	12.7	11.3
Other non-antipseudomonal agents	6.1	8.3	3.2	8.1	3.4	2.7	5.7	5.3	6.7	3.3	3.5	4.4
Total	180.3	200.7	178.2	179.1	153.4	157.4	182.1	167.9	154.2	152.8	157.8	162.5

MRSA, methicillin-resistant *Staphylococcus aureus*

Supplemental Table S2. Monthly duration of each antibiotic treatment, median days (IQR)

	Antipseudomonal agents		Anti-MRSA agents		Other antibiotics	
	n	Median days (IQR)	n	Median days (IQR)	n	Median days (IQR)
May-17	231	6 (3–8)	90	4 (1–8)	886	3 (1–4)
Jun-17	211	6 (3–8)	109	3 (1–7)	930	3 (1–4)
Jul-17	215	6 (3–8)	94	4 (1–9)	922	3 (1–4)
Aug-17	204	6 (4–10)	91	5 (2–10)	985	3 (1–4)
Sep-17	211	6 (3–8)	63	6 (2–10)	933	3 (1–4)
Oct-17	209	6 (4–9)	82	4 (1–10)	897	3 (1–4)
Nov-17	212	6 (3–8)	65	4 (1–9)	903	3 (1–4)
Dec-17	196	6 (3–8)	86	4 (1–9)	958	2 (1–4)
May-18	176	6 (4–9)	68	3 (1–6)	876	3 (1–4)
Jun-18	150	6 (3–9)	50	6 (3–14)	937	2 (1–4)
Jul-18	159	6 (4–8)	75	5 (1–12)	939	3 (1–4)
Aug-18	230	6 (3–8)	114	3 (1–7)	1051	2 (1–4)
Sep-18	202	5 (3–8)	115	5 (2–8)	846	3 (1–4)
Oct-18	214	6 (3–8)	99	3 (1–7)	942	3 (1–4)
Nov-18	187	5 (3–8)	75	3 (1–6)	878	3 (2–4)
Dec-18	244	5 (3–8)	105	3 (1–6)	898	2 (1–4)
May-19	201	5 (3–8)	83	3 (1–7)	825	3 (1–4)
Jun-19	183	5 (3–8)	88	3 (1–7)	896	2 (1–4)
Jul-19	233	6 (3–8)	126	3 (1–7)	1027	2 (1–4)
Aug-19	209	6 (3–9)	84	4 (1–9)	947	2 (1–4)
Sep-19	191	5 (3–8)	81	4 (1–11)	894	2 (1–4)
Oct-19	164	5 (4–8)	83	6 (3–10)	910	2 (1–4)
Nov-19	164	5 (3–8)	85	3 (1–9)	893	2 (1–4)
Dec-19	214	5 (3–8)	97	3 (1–7)	910	2 (1–4)

MRSA, methicillin-resistant *Staphylococcus aureus*

Supplemental Table S3. Monthly duration of each antibiotic treatment, mean days (SD)

	Antipseudomonal agents		Anti-MRSA agents		Other antibiotics	
	n	Mean days (SD)	N	Mean days (SD)	n	Mean days (SD)
May-17	231	7 (6)	90	7 (9)	886	4 (4)
Jun-17	211	7 (5)	109	5 (6)	930	3 (4)
Jul-17	215	6 (5)	94	7 (7)	922	4 (5)
Aug-17	204	8 (8)	91	8 (10)	985	4 (4)
Sep-17	211	7 (6)	63	7 (7)	933	4 (6)
Oct-17	209	7 (7)	82	8 (9)	897	3 (4)
Nov-17	212	7 (4)	65	8 (12)	903	4 (5)
Dec-17	196	7 (5)	86	7 (8)	958	3 (4)
May-18	176	7 (6)	68	6 (9)	876	4 (6)
Jun-18	150	7 (6)	50	13 (31)	937	4 (4)
Jul-18	159	7 (5)	75	9 (10)	939	4 (6)
Aug-18	230	7 (5)	114	6 (8)	1051	4 (4)
Sep-18	202	7 (6)	115	7 (6)	846	3 (3)
Oct-18	214	6 (5)	99	5 (7)	942	3 (4)
Nov-18	187	6 (7)	75	6 (9)	878	4 (5)
Dec-18	244	6 (5)	105	5 (6)	898	3 (3)
May-19	201	6 (8)	83	5 (6)	825	4 (6)
Jun-19	183	6 (5)	88	7 (11)	896	4 (4)
Jul-19	233	7 (5)	126	5 (5)	1027	4 (4)
Aug-19	209	7 (5)	84	6 (8)	947	3 (4)
Sep-19	191	6 (5)	81	8 (9)	894	3 (4)
Oct-19	164	7 (5)	83	8 (9)	910	3 (4)
Nov-19	164	6 (4)	85	6 (8)	893	4 (4)
Dec-19	214	6 (6)	97	7 (10)	910	3 (4)

MRSA, methicillin-resistant *Staphylococcus aureus*

Supplemental Table S4. Monthly antibiotic cost (US$).

	Antibiotic cost
May-17	69,154
Jun-17	71,750
Jul-17	75,668
Aug-17	89,396
Sep-17	82,674
Oct-17	90,216
Nov-17	92,545
Dec-17	77,762
May-18	69,621
Jun-18	82,613
Jul-18	70,826
Aug-18	66,672
Sep-18	70,821
Oct-18	80,832
Nov-18	79,289
Dec-18	71,281
May-19	65,172
Jun-19	52,725
Jul-19	61,423
Aug-19	79,927
Sep-19	82,294
Oct-19	83,760
Nov-19	87,603
Dec-19	87,081

Supplemental Table S5. Susceptibility categorization (S/I/R) of *Pseudomonas aeruginosa* to each antibiotic per month

		PIPC			CFPM			MEPM			AMK			LVFX		
	n	S	I	R	S	I	R	S	I	R	S	I	R	S	I	R
May-17	18	15	1	2	16	0	2	16	1	1	18	0	0	15	2	1
Jun-17	14	11	2	1	13	0	1	12	0	2	14	0	0	12	0	2
Jul-17	17	16	1	0	17	0	0	13	1	3	17	0	0	16	1	0
Aug-17	12	11	0	1	12	0	0	12	0	0	12	0	0	12	0	0
Sep-17	12	12	0	0	11	1	0	10	1	1	12	0	0	12	0	0
Oct-17	11	10	0	1	9	2	0	10	0	1	11	0	0	10	0	1
Nov-17	16	14	0	2	14	0	2	14	0	2	16	0	0	14	1	1
Dec-17	12	11	0	1	12	0	0	12	0	0	12	0	0	12	0	0
May-18	15	14	0	1	14	0	1	15	0	0	15	0	0	12	0	3
Jun-18	6	4	1	1	5	0	1	5	0	1	6	0	0	6	0	0
Jul-18	12	12	0	0	11	1	0	11	0	1	12	0	0	12	0	0
Aug-18	13	13	0	0	13	0	0	12	0	1	13	0	0	13	0	0
Sep-18	10	8	1	1	8	2	0	9	1	0	10	0	0	9	0	1
Oct-18	21	19	1	1	19	2	0	20	1	0	21	0	0	20	0	1
Nov-18	14	12	2	0	13	1	0	13	1	0	14	0	0	14	0	0
Dec-18	15	13	2	0	14	1	0	14	1	0	15	0	0	15	0	0
May-19	17	14	1	2	14	3	0	14	1	2	17	0	0	15	0	2
Jun-19	15	12	2	1	12	3	0	13	0	2	14	1	0	14	1	0
Jul-19	16	16	0	0	16	0	0	14	0	2	16	0	0	15	0	1
Aug-19	16	14	0	2	13	1	2	15	0	1	15	1	0	14	0	2
Sep-19	23	21	1	1	22	1	0	23	0	0	23	0	0	23	0	0
Oct-19	20	18	2	0	19	1	0	20	0	0	20	0	0	19	1	0
Nov-19	13	13	0	0	13	0	0	13	0	0	12	1	0	13	0	0
Dec-19	15	14	0	1	13	2	0	14	1	0	15	0	0	15	0	0

S, susceptible; I, intermediate; R, resistant; PIPC, piperacillin; CFPM, cefepime; MEPM, meropenem; AMK, amikacin; LVFX, levofloxacin

Supplemental Table S6. Demographic characteristics of patients with bacteremia

	Baseline (n = 421)	Intervention (n = 364)	Post-intervention (n = 437)	*p*
Age ≥65 years, n (%)	244 (61)	230 (65)	253 (63)	0.86
Male sex, n (%)	269 (67)	241 (68)	272 (67)	0.54
Admission to the intensive care unit, n (%)	83 (21)	61 (17)	73 (18)	0.36

Supplemental Table S7. Monthly length of hospital stay for patients who received antibiotics, median days (IQR).

	Antipseudomonal agents		Anti-MRSA agents		Other antibiotics	
	n	Median (IQR)	n	Median (IQR)	n	Median (IQR)
May-17	130	24 (14–62)	51	36 (18–82)	686	12 (7–24)
Jun-17	119	27 (15–48)	51	40 (25–74)	741	12 (6–24)
Jul-17	125	29 (16–53)	48	47 (22–78)	704	12 (6–22)
Aug-17	114	35 (17–63)	52	46 (31–72)	742	12 (6–22)
Sep-17	112	32 (17–52)	41	52 (27–80)	718	11 (6–23)
Oct-17	119	28 (15–58)	53	43 (27–78)	686	13 (6–24)
Nov-17	112	23 (15–39)	30	31 (22–62)	697	11 (6–23)
Dec-17	121	26 (14–53)	48	53 (28–77)	712	11 (6–22)
May-18	104	31 (16–60)	39	40 (21–64)	691	13 (6–22)
Jun-18	89	29 (16–50)	27	44 (29–67)	731	11 (6–22)
Jul-18	96	29 (15–52)	40	61 (23–104)	753	12 (6–21)
Aug-18	125	31 (18–49)	54	43 (30–64)	837	11 (6–22)
Sep-18	115	35 (19–60)	60	49 (30–67)	647	13 (7–24)
Oct-18	111	26 (16–63)	43	34 (21–66)	718	13 (7–24)
Nov-18	103	27 (13–48)	40	49 (23–85)	678	12 (6–22)
Dec-18	130	35 (16–61)	51	43 (30–70)	701	12 (6–24)
May-19	128	31 (16–54)	49	41 (24–68)	611	13 (6–24)
Jun-19	111	29 (15–43)	58	35 (26–67)	674	11 (6–21)
Jul-19	137	24 (13–44)	67	44 (26–69)	776	12 (6–21)
Aug-19	124	27 (16–52)	42	46 (27–71)	725	12 (6–21)
Sep-19	110	32 (16–56)	41	38 (19–69)	667	12 (6–22)
Oct-19	97	26 (12–55)	44	59 (32–92)	694	11 (6–22)
Nov-19	96	24 (12–47)	31	47 (28–69)	701	11 (6–22)
Dec-19	133	23 (12–48)	52	45 (19–73)	705	12 (6–22)

MRSA, methicillin-resistant *Staphylococcus aureus.*

Supplemental Table S8. Monthly length of hospital stay for patients who received antibiotics, mean days (SD)

	Antipseudomonal agents		Anti-MRSA agents		Other antibiotics	
	n	Mean (SD)	n	Mean (SD)	n	Mean (SD)
May-17	130	48 (53)	51	64 (92)	686	22 (32)
Jun-17	119	42 (58)	51	57 (54)	741	22 (35)
Jul-17	125	48 (74)	48	60 (62)	704	20 (37)
Aug-17	114	51 (57)	52	63 (60)	742	19 (26)
Sep-17	112	42 (39)	41	58 (43)	718	19 (24)
Oct-17	119	42 (40)	53	61 (55)	686	20 (26)
Nov-17	112	31 (25)	30	44 (33)	697	20 (24)
Dec-17	121	39 (39)	48	58 (42)	712	19 (24)
May-18	104	47 (47)	39	47 (34)	691	20 (27)
Jun-18	89	39 (36)	27	53 (34)	731	19 (26)
Jul-18	96	42 (44)	40	81 (71)	753	19 (24)
Aug-18	125	43 (49)	54	60 (63)	837	18 (22)
Sep-18	115	45 (37)	60	58 (43)	647	21 (26)
Oct-18	111	46 (44)	43	49 (38)	718	21 (28)
Nov-18	103	44 (65)	40	71 (73)	678	21 (36)
Dec-18	130	49 (55)	51	57 (50)	701	20 (26)
May-19	128	44 (49)	49	53 (47)	611	22 (32)
Jun-19	111	41 (49)	58	60 (66)	674	18 (25)
Jul-19	137	32 (31)	67	50 (40)	776	18 (24)
Aug-19	124	39 (35)	42	57 (45)	725	18 (23)
Sep-19	110	42 (39)	41	50 (40)	667	19 (22)
Oct-19	97	39 (38)	44	69 (48)	694	20 (27)
Nov-19	96	37 (39)	31	63 (57)	701	19 (23)
Dec-19	133	39 (43)	52	55 (42)	705	19 (21)

MRSA, methicillin-resistant *Staphylococcus aureus*
